# The Nuclear Mitotic Apparatus (NuMA) Protein: A Key Player for Nuclear Formation, Spindle Assembly, and Spindle Positioning

**DOI:** 10.3389/fcell.2021.653801

**Published:** 2021-04-01

**Authors:** Tomomi Kiyomitsu, Susan Boerner

**Affiliations:** Cell Division Dynamics Unit, Okinawa Institute of Science and Technology Graduate University, Onna-son, Japan

**Keywords:** NuMA, spindle, nuclear formation, dynein, Ran-GTP

## Abstract

The nuclear mitotic apparatus (NuMA) protein is well conserved in vertebrates, and dynamically changes its subcellular localization from the interphase nucleus to the mitotic/meiotic spindle poles and the mitotic cell cortex. At these locations, NuMA acts as a key structural hub in nuclear formation, spindle assembly, and mitotic spindle positioning, respectively. To achieve its variable functions, NuMA interacts with multiple factors, including DNA, microtubules, the plasma membrane, importins, and cytoplasmic dynein. The binding of NuMA to dynein via its N-terminal domain drives spindle pole focusing and spindle positioning, while multiple interactions through its C-terminal region define its subcellular localizations and functions. In addition, NuMA can self-assemble into high-ordered structures which likely contribute to spindle positioning and nuclear formation. In this review, we summarize recent advances in NuMA’s domains, functions and regulations, with a focus on human NuMA, to understand how and why vertebrate NuMA participates in these functions in comparison with invertebrate NuMA-related proteins.

## Introduction

**FIGURE 1:**
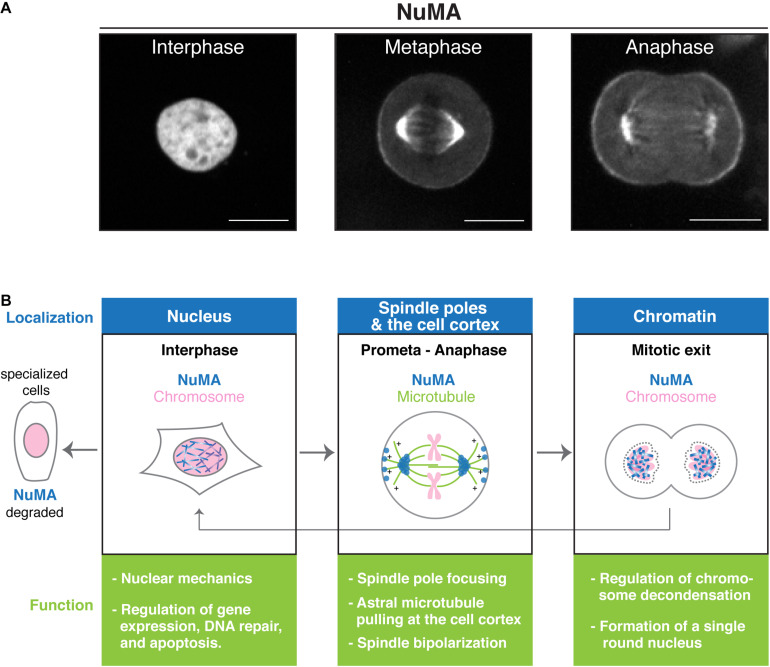
Nuclear mitotic apparatus (NuMA’s) functions during the cell cycle. **(A)** Images of endogenous NuMA fused with mClover in human HCT116 cells (Okumura et al., 2018). NuMA accumulates in the nucleus in interphase, but mainly localizes at the spindle poles in metaphase. NuMA is also targeted to the cell cortex near the spindle poles during metaphase, and the level increases during anaphase (Collins et al., 2012; Kiyomitsu and Cheeseman, 2013; Kotak et al., 2013; Seldin et al., 2013; Zheng et al., 2014). Scale bars = 10 μm. **(B)** Schematic showing how NuMA (blue) dynamically changes its subcellular localization during the cell cycle. Chromosomes and microtubules are shown in pink and green, respectively. Key functions of NuMA at these locations are summarized.

The nuclear mitotic apparatus (NuMA) protein was initially identified as a non-histone chromatin protein in human cell lines (Lydersen et al., 1980) and named after its localization pattern to both the interphase nucleus and mitotic spindle poles (Lydersen and Pettijohn, 1980; Figure 1A). Since NuMA’s dynamic translocation from the nucleus to the spindle poles was different from previously characterized nuclear components, NuMA was regarded as a novel class of nuclear protein, involved in both mitosis and nuclear reformation (Compton and Cleveland, 1994). Over the last 40 years, NuMA has been extensively studied in mammalian cultured cells, *Xenopus* egg extracts, and other vertebrate models (Cleveland, 1995; Sun and Schatten, 2006; Radulescu and Cleveland, 2010). One of the key findings early on was that NuMA interacts with cytoplasmic dynein to tether microtubules to spindle poles (Merdes et al., 1996). Later studies confirmed and expanded upon this result, positioning NuMA as a mitotic dynein adaptor, as described below.

Another important finding was that NuMA interacts with leucine/glycine/asparagine-repeat-containing protein (LGN) to form the evolutionarily conserved NuMA/LGN/Gαi complex at the mitotic cell cortex (Du and Macara, 2004). This study led to the discovery that NuMA plays a conserved role at the mitotic cell cortex for spindle positioning like *C. elegans* LIN-5 (Lorson et al., 2000; Srinivasan et al., 2003) and *Drosophila* Mud [Bowman et al., 2006; Izumi et al., 2006; Siller et al., 2006; The first publication is Lorson et al. (2000)]. Building on this, and other pioneering work on asymmetric cell division in *C. elegans* and *Drosophila* (Galli and van den Heuvel, 2008; Gonczy, 2008; Siller and Doe, 2009), further studies have established the conceptual framework that cortical cues converge on a conserved ternary complex, NuMA/LGN/Gαi in vertebrates, Lin-5/GPR-1/2/Gα in *C. elegans*, and Mud/Pins/Gα in *Drosophila*, that recruits and activates dynein to position the spindle in asymmetric division (Lechler and Fuchs, 2005; Poulson and Lechler, 2010; Morin and Bellaiche, 2011; Williams et al., 2011). In symmetrically-dividing vertebrate cells, the NuMA/LGN/Gαi complex is also involved in recruiting dynein and controlling spindle position and orientation (Figure 1A; Woodard et al., 2010; Peyre et al., 2011; Collins et al., 2012; Kiyomitsu and Cheeseman, 2012; Kotak et al., 2012; Kiyomitsu, 2019).

Interestingly, the cortical function seems to be most conserved in NuMA-related proteins. For example, LIN-5 and Mud localize at both spindle poles and the cell cortex, but only serve an essential function at the cell cortex for spindle positioning and are non-essential for bipolar spindle assembly (Lorson et al., 2000; Bowman et al., 2006; Izumi et al., 2006; Siller et al., 2006). In addition, yeast Num1 was recently proposed as a functional homolog of NuMA based on its functional similarities at the cell cortex (Greenberg et al., 2018). Intriguingly, plants lack a homolog of NuMA, as well as cytoplasmic dynein (Yamada and Goshima, 2017), suggesting that plants have developed alternative mechanisms to control nuclear formation, spindle assembly and positioning.

In the last 10 years, many functional domains of human NuMA were identified, providing useful information to understand NuMA’s functions and regulations at the molecular level. In this review, we focus on vertebrate NuMA because several features, such as nuclear localization, appear to be specific to this group. We begin with an overview of the localization and the structural domains of the human NuMA protein, and then discuss how vertebrate NuMA participates in spindle assembly, spindle positioning and nuclear formation.

## Localization and an Overall Structure of NuMA

In cultured human cells, NuMA accumulates in the nucleus in interphase and at the spindle poles and cell cortex during mitosis (Figure 1A). NuMA’s spindle-pole localization is most likely conserved in all cell types, including meiotic cells (Taimen et al., 2004; Alvarez Sedo et al., 2011; Kolano et al., 2012), but its nuclear and cortical localization may vary in different developmental contexts: nuclear NuMA is degraded in some specialized cells, such as smooth and skeletal muscle fibers (Merdes and Cleveland, 1998; Figure 1B left). Furthermore, cortical NuMA targeting is differentially regulated between symmetric and asymmetric division in mouse epidermal cells (Poulson and Lechler, 2010).

**FIGURE 2:**
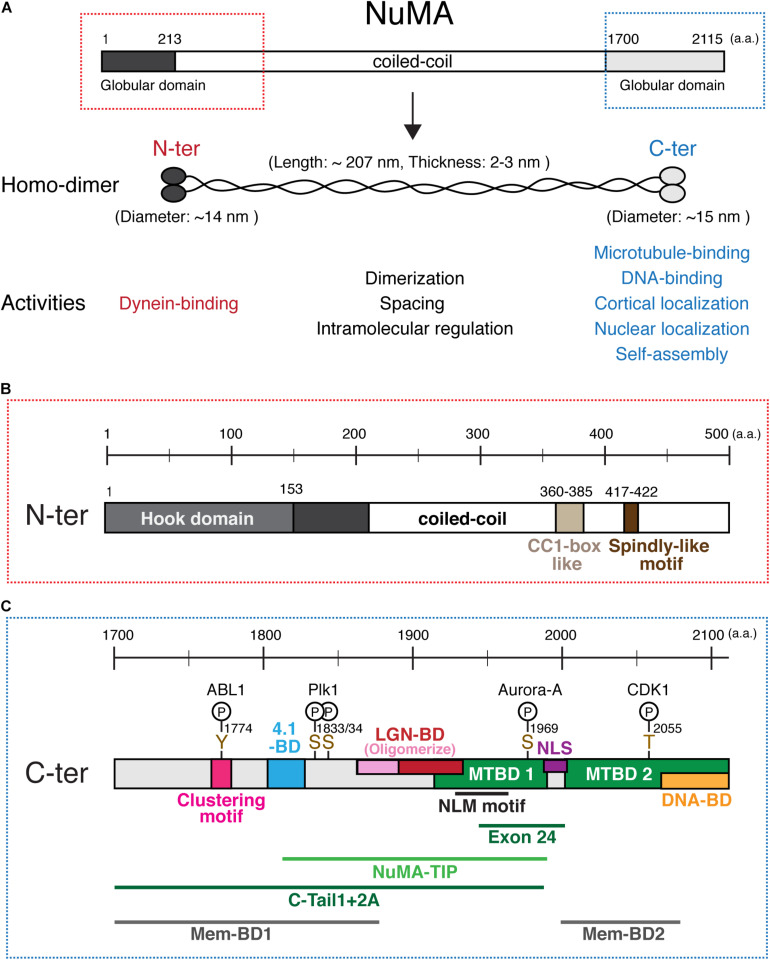
NuMA’s domain structure. **(A)** Diagrams of full length NuMA. Human NuMA isoform 1 (NP_006176) consists of 2,115 amino acids (a.a.) (Yang et al., 1992), whereas NuMA isoform 2 (NP_001273490) is lacking 14 a.a. at the 1,539–1,552 region (Compton et al., 1992). In this review, we refer to isoform 1. NuMA forms a homodimer through its central coiled-coil region. The central rod-shaped structure has an average length of ∼207 nm and a thickness of ∼2–3 nm, while N-terminal and C-terminal globular structures show a diameter of ∼14 and ∼15 nm, respectively (Harborth et al., 1995). **(B)** Domains in the N-terminal region. **(C)** Domains, motifs, phosphorylation sites, and functional regions in the C-terminal region of NuMA. See the text and Table 1 for details. NuMA^1944–2003^, corresponding to either exon 22 for mice or exon 24 for human, was deleted in Silk et al. (2009); Kolano et al. (2012), and Tsuchiya et al. (2021), instead of depleting the complete MTBD1.

Human NuMA is a large (∼238 kDa) protein that consists of N-terminal and C-terminal globular domains and a central long coiled-coil domain (Compton et al., 1992; Yang et al., 1992; Figure 2A). Full length NuMA expressed in *E. coli* forms a homo-dimer using the coiled-coil region (Harborth et al., 1995; Forth et al., 2014). Purified NuMA shows a long rod-shaped structure that has globular ends and a central long (∼210 nm) α-helical domain that appears more or less flexible (Harborth et al., 1995, 1999; Figure 2A). The globular domains interact with many factors, whereas the central region has structural and likely intramolecular regulatory roles as described below.

## Dynein-Binding Domains in the N-Terminal Region of NuMA

The N-terminal region is required to interact with cytoplasmic dynein and dynactin complexes during mitosis (Kotak et al., 2012). A recent structural study revealed that NuMA^1–153^ contains a Hook domain that directly interacts with dynein light intermediate chain (LIC) 1 and 2 (Renna et al., 2020; Figure 2B; all domains are listed in Table 1). The authors also identified a CC1-box like motif, NuMA^360–385^, adjacent to the Hook domain which forms part of the binding interface between NuMA and LIC (Renna et al., 2020; Figure 2B). In addition, NuMA^417–422^ contains a Spindly-like motif which is well conserved in vertebrates (Okumura et al., 2018; Tsuchiya et al., 2021), and may interact with the dynactin point-end complex (Gama et al., 2017; Lee et al., 2020). As NuMA^1–505^, but not NuMA^1–413^ and NuMA^214–705^, is sufficient for dynein recruitment to the cell cortex (Okumura et al., 2018), multiple interaction sites in the N-terminal region appear to be important to stably interact with the dynein-dynactin complex during mitosis. These studies indicate that NuMA acts as a dynein activating adaptor (Lee et al., 2020), but in contrast to other established dynein adaptors such as BICD2 (McKenney et al., 2014; Schlager et al., 2014), its ability to form a ternary complex with purified dynein and dynactin and activate dynein motility *in vitro* has not been shown; post translational modifications may be required to form dynein–dynactin–NuMA complexes during mitosis.

**TABLE 1:** A summary of NuMA’s domain and modifications.

Region (a.a.)	Domain and modification	References
1–153	Hook domain that interacts with LIC 1 and 2	Renna et al., 2020
1–213	Globular domain	Radulescu and Cleveland, 2010
1–505	Sufficient for cortical dynein recruitment	Okumura et al., 2018
360–385	CC1-box like motif	Renna et al., 2020
417–422	Spindly-like motif	Okumura et al., 2018; Tsuchiya et al., 2021
199–432	Dimerization	Harborth et al., 1995
670–1700	Dimerization	Harborth et al., 1995
1–400	Dimerization	Forth et al., 2014
Coiled-coil region (706–1699)	Required for spindle pulling force generation. Inhibits chromatin binding during anaphase and promotes the formation of a single round nucleus	Okumura et al., 2018; Serra-Marques et al., 2020
1699–1876	Membrane binding region (Mem-BD) 1	Kotak et al., 2014
1701–1725	Cleavage site during apoptosis	Gueth-Hallonet et al., 1997
1701–1981	C-tail1 + 2A: sufficient for minus-end targeting	Hueschen et al., 2017
1768–1777	Clustering domain	Okumura et al., 2018
1788–1925	Sufficient for metaphase cortical localization	Seldin et al., 2013
1802–1824	4.1 protein binding region	Mattagajasingh et al., 2009
1811–1985	NuMA-TIP	Seldin et al., 2016
1861–1928	Longer binding region of LGN^7–367^	Pirovano et al., 2019
1900–1926	Minimal binding region of LGN	Zhu et al., 2011b
1914–1985	Microtubule binding domain (MTBD) 1	Du et al., 2002
1922–1957	NLM motif	Siller et al., 2006
1944–2003	Human exon 24 (=mouse exon 22)	Silk et al., 2009; Gallini et al., 2016
1996–2074	Membrane binding region (Mem-BD) 2	Zheng et al., 2014
1988–2005	NLS sequence	Tang et al., 1994; Chang et al., 2017
2002–2115	Microtubule binding domain (MTBD) 2	Gallini et al., 2016; Chang et al., 2017
2058–2115	DNA binding domain	Rajeevan et al., 2020
Y1774	Phosphorylation residue by ABL1	Matsumura et al., 2012
SS1833/34	Phosphorylation residues by Plk1	Kettenbach et al., 2011
S1969	Phosphorylation residue by Aurora-A kinase (at spindle pole)	Gallini et al., 2016; Kotak et al., 2016
T2055	Phosphorylation residue by CDK (during metaphase)	Compton and Luo, 1995; Kotak et al., 2013
Full length NuMA	MARs (DNA sequence) binding	Luderus et al., 1994

On the other hand, the interphase role of the N-terminal region of NuMA is not clear. Although both N-terminal and C-terminal globular domains contain several S/TPXX motifs which are supposed to contribute to DNA binding (Suzuki, 1989), the N-terminal NuMA fragments do not show clear DNA or chromatin binding compared to NuMA’s C-terminal (Serra-Marques et al., 2020). However, the N-terminal domain may contribute to the lattice formation of NuMA oligomers in the nucleus (Gueth-Hallonet et al., 1998; Harborth et al., 1999), as described below.

## The Central Long Coiled-Coil Domain of NuMA

Electron micrographs of recombinant human NuMA indicate that the central region forms a long flexible rod-shaped structure (Harborth et al., 1995; Figure 2A). One of most important functions of the central region is homo-dimerization. *In vitro* studies suggest that either the N-terminal (199–432 or 1–400 a.a) or C-terminal part (670–1700 a.a.) of the central coiled-coil is sufficient to form a homo-dimer (Harborth et al., 1995; Forth et al., 2014). Dimerization is most likely critical for most NuMA functions, including dynein binding via its N-terminal Hook domain (Renna et al., 2020) and microtubule cross-linking activities via the C-terminal domain (Forth et al., 2014).

The predicted long coiled-coil region of vertebrate NuMA proteins, which exceeds 1,000 a.a. in length, is likely to perform other important functions. Transient overexpression of wild type NuMA in HeLa cells induced a regular nuclear lattice structure which has quasi-hexagonal organization (Gueth-Hallonet et al., 1998). Interestingly, an addition or deletion in the coiled-coil domain changed the spacing of the hexagons, suggesting that the central coiled-coil defines the length of the nuclear lattice structure (Gueth-Hallonet et al., 1998; Harborth et al., 1999).

Recently, Serra-Marques et al. (2020) found that the long coiled-coil is also required for the formation of a single, round nucleus, and that this role is independent from NuMA’s role in spindle formation. In addition, the authors revealed that a small portion of the coiled-coil^213–705^ is sufficient to prevent NuMA’s C-terminal region from binding chromosomes during late metaphase and anaphase (Serra-Marques et al., 2020).

At the mitotic cell cortex, the coiled-coil region of NuMA^706–1699^ is required to generate proper spindle pulling forces when NuMA constructs are targeted to the membrane during mitosis (Okumura et al., 2018). However, the coiled-coil region is dispensable for bipolar spindle formation (Hueschen et al., 2017). At the cell cortex, the long coiled-coil may be used to separate dynein from the actin-rich cell cortex and/or to increase the efficiency of astral microtubule capture by cortical NuMA-dynein complexes during mitosis (Kiyomitsu, 2019).

## Microtubule-Binding Domains in NuMA’s C-Terminal Region

**FIGURE 3:**
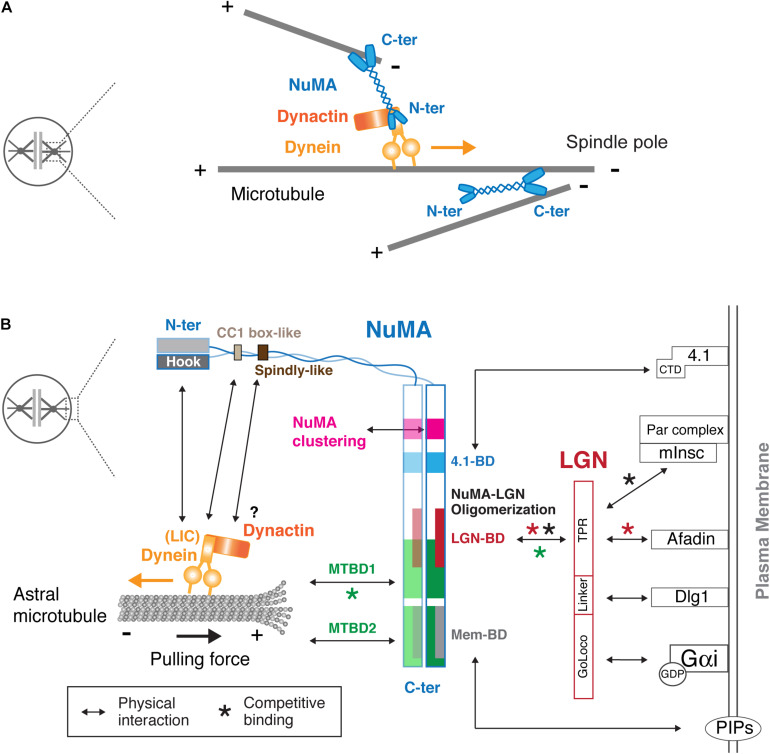
Physical interaction map of NuMA at the spindle pole and the mitotic cell cortex. **(A)** Models of the spindle pole focusing function of NuMA. Using two microtubule-binding C-terminal globular domains, the NuMA homodimer bundles and crosslinks microtubules around the spindle pole region (Radulescu and Cleveland, 2010; Forth et al., 2014). In addition, NuMA recognizes the minus-ends of spindle microtubules and recruits the dynein-dynactin complex, which transports NuMA-bound spindle microtubules toward poles, resulting in microtubule focusing at the poles (Hueschen et al., 2017). See text for details. **(B)** NuMA interacts with the cortical proteins and plasma membrane through its C-terminal region, whereas it binds to dynein and dynactin via its N-terminal region. Arrows indicate physical interactions. As indicated by red asterisks, NuMA competes with Afadin for LGN binding. In addition, NuMA competes with mInsc for LGN binding during asymmetric cell division (black asterisks). The MTBD1 overlaps LGN–BD, and thus LGN binding to NuMA inhibits the microtubule binding activity of MTBD1 (green asterisks). LGN consists of TPR, Linker and GoLoco motifs, and each motif interacts with Afadin (Carminati et al., 2016), Dlg1 (Saadaoui et al., 2014), and the GDP-bound form of Gαi (Jia et al., 2012), respectively. NuMA/LGN/Gαi constitutes a conserved core pathway for cortical dynein recruitment. The C-terminal domain (CTD) of band 4.1 proteins interacts with the NuMA C-terminal domain, and is sufficient to rescue cortical NuMA enrichment in anaphase in LGN and 4.1 co-depleted cells (Kiyomitsu and Cheeseman, 2013). See text for details.

The C-terminal globular region of NuMA^1700–2115^ contains several important domains that determine its localization and function (Figure 2C). First, this region contains two microtubule binding domains (MTBDs); here we refer to them as MTBD1^1914–1985^ (Du et al., 2002) and MTBD2^2002–2115^ (Gallini et al., 2016; Chang et al., 2017; Figure 2C). Although MTBD1 has weaker microtubule-binding affinity than MTBD2 *in vitro* (Chang et al., 2017), MTBD1 contains the conserved NuMA–LIN-5–Mud (NLM) motif^1922–1957^ (Siller et al., 2006) and acts to establish and maintain spindle-pole focusing (Figure 3A) in mouse fibroblasts (Silk et al., 2009), mouse meiosis I spindle (Kolano et al., 2012), and human HCT116 cells (Tsuchiya et al., 2021).

In some cells, such as mouse keratinocytes, MTBD1 is not required for spindle pole focusing, but is required for spindle orientation (Seldin et al., 2016). Full length NuMA accumulates at the minus-end of microtubules, and the NuMA^1701–1981^ fragment, called C-Tail1 + 2A, is sufficient for minus-end recognition (Hueschen et al., 2017). In addition, NuMA’s dimerized C-terminal fragment that crosslinks two parallel microtubules tends to move in the minus-end direction under forces (Forth et al., 2014). However, intriguingly, the NuMA^1811–1985^ fragment, called NuMA-TIP, appears to preferentially accumulate at the curling microtubule ends, and can remain attached to the depolymerizing microtubule plus-end (Seldin et al., 2016). The authors propose that this unique property of MTBD1 may be important to facilitate the interaction of astral microtubule plus-ends with NuMA at the cell cortex during spindle orientation (Seldin et al., 2016). However, in human cells, MTBD2, but not MTBD1, is required for spindle pulling activity when NuMA constructs are targeted to the cell cortex (Okumura et al., 2018). Interestingly, MTBD2 can bind both the microtubule lattice and tubulin dimers (Pirovano et al., 2019), indicating that MTBD2 may act not only for astral microtubule binding, but also for regulating plus-end dynamics of astral microtubules during the cortical pulling-force generation. How and when the MTBDs come into play during spindle pole focusing and orientation likely depends on the cellular context (Borgal and Wakefield, 2018).

## Cortical Targeting Domains in NuMA’s C-Terminal Region

NuMA’s C-terminal domain also defines its cortical localization during mitosis by binding to LGN, band 4.1 proteins and the plasma membrane. Whereas LGN is targeted to the cell cortex by binding to GDP-bound Gαi through its C-terminal GoLoco motif, LGN binds and links NuMA to the cell cortex using its N-terminal TPR motif (Figure 3B). LGN and Gαi are indispensable for cortical localization of NuMA in metaphase (Du and Macara, 2004; Woodard et al., 2010; Kiyomitsu and Cheeseman, 2012; Kotak et al., 2012). Zhu et al. (2011b) identified the NuMA^1900–1926^ peptide as the minimal region required to bind to the inner groove of LGN^TPR^. This LGN-binding domain partially overlaps with MTBD1 (Figure 2C), and LGN binding thus inhibits the microtubule binding activity of NuMA *in vitro* (Du et al., 2002; Figure 3, green asterisk). Recently, Pirovano et al. (2019) revealed that a longer NuMA^1861–1928^ region forms a hetero-hexamer with LGN^7–367^, in which an extended NuMA^1861–1880^ region hooks onto an adjacent LGN to form oligomers. Consistently, expression of a longer NuMA^1788–1925^ fragment is targeted to the LGN-localizing metaphase cell cortex, whereas a shorter one, NuMA^1892–1925^, is not (Seldin et al., 2013).

However, given the complicated cortical protein network, additional mechanisms might contribute to cortical NuMA–LGN targeting and stability. In fact, it has been shown that cortical NuMA–LGN is affected by disrupting cortical actin networks (Kaji et al., 2008; Carminati et al., 2016) or their regulators (Machicoane et al., 2014; Kschonsak and Hoffmann, 2018). Recently, Carminati et al. (2016) demonstrated that F-actin binding protein Afadin, which also binds LGN competitively with NuMA, is required to facilitate NuMA–LGN complex formation at the metaphase cell cortex (Figure 3B). On the other hand, Dlg1, that directly interacts with the phosphorylated LGN linker region (Zhu et al., 2011a) is also required for cortical LGN and NuMA localization (Saadaoui et al., 2014).

In asymmetrically dividing epithelial or mammary stem cells, the situation is more complex. Par3-binding mInsc (mammalian homolog of Inscuteable) and NuMA compete for binding to LGN^TPR^, with mInsc showing a more than fivefold higher affinity (Culurgioni et al., 2011; Yuzawa et al., 2011; Zhu et al., 2011b). Previously, mInsc-bound LGN-Gαi was supposed to be transferred to NuMA, but Cukurgioni et al. recently reported that the Inscuteable-LGN tetramer is so stable that LGN cannot be dissociated from Inscuteable by NuMA (Culurgioni et al., 2018). The authors proposed that the Inscuteable-LGN tetramer generates a localized pool of Gαi-GTP molecules, which upon GTP-hydrolysis recruits a distinct population of LGN that subsequently recruits NuMA and dynein to orient the spindle (Culurgioni et al., 2018).

Although the LGN pathways are critical for cortical NuMA localization during metaphase in both symmetric and asymmetric divisions (Morin and Bellaiche, 2011), recent studies revealed that NuMA can be targeted to the anaphase cell cortex independently of LGN in symmetrically dividing mammalian cells (Collins et al., 2012; Kiyomitsu and Cheeseman, 2013; Kotak et al., 2013; Seldin et al., 2013; Zheng et al., 2014). Band 4.1 proteins link the plasma membrane to the actin cytoskeleton (Baines et al., 2014), and interact with NuMA via their C-terminal domain (CTD) and NuMA’s 4.1-binding domain (1802–1824) (Mattagajasingh et al., 2009; Figure 3B). This NuMA-4.1 interaction appears to be important for the cortical stability of NuMA in metaphase keratinocytes (Seldin et al., 2013), but also has an important role for anaphase NuMA localization: double depletion of LGN and 4.1 proteins eliminates cortical NuMA in anaphase human cells (Kiyomitsu and Cheeseman, 2013). Importantly, this phenotype was rescued by the expression of the membrane-targeted C-terminal domain (CTD) of band 4.1 (Kiyomitsu and Cheeseman, 2013), which does not bind to actin, suggesting that 4.1 proteins contribute to anaphase NuMA localization independently of LGN and cortical actin structures. How 4.1 proteins regulate NuMA remains unclear; one possibility is that they increase cortical NuMA retention by linking it with the cell cortex and/or transferring NuMA to the plasma membrane (as described below). In addition to these cortical proteins, other proteins may be involved in cortical NuMA recruitment in developmental contexts. For example, NuMA’s C-terminal region interacts with Disheveled, which controls spindle orientation during Zebrafish gastrulation (Segalen et al., 2010).

Importantly, NuMA can directly interact with the plasma membrane. The C-terminal region contains two membrane-binding domains, NuMA^1699–1876^ (Kotak et al., 2014) and NuMA^1996–2074^ (Zheng et al., 2014), which overlap with 4.1-BD and MTBD2, respectively, (Figure 2C) and are referred to as Mem-BD1 and 2 in this review (Figure 2C). Both membrane-binding domains preferentially bind to phosphorylated forms of phosphatidylinositol (PIPs) and are required for efficient cortical accumulation of NuMA (Kotak et al., 2014; Zheng et al., 2014). Mem-BD2, which partially overlaps with the DNA-binding domain (Figure 2C), is required for proper chromosome separation during anaphase (Zheng et al., 2014).

## DNA-Binding Domain in NuMA’s C-Terminal Region

In contrast to invertebrate NuMA-related proteins, vertebrate NuMA proteins analyzed so far localize in the nucleus (Lydersen and Pettijohn, 1980; Compton et al., 1992; Yang et al., 1992; Merdes et al., 1996). Previously, human NuMA was reported to interact with defined DNA sequences called matrix attached regions (MARs) *in vitro* (Luderus et al., 1994). Recently, two studies demonstrated that the C-terminal region of human NuMA interacts with DNA *in vitro* and chromatin in cells (Rajeevan et al., 2020; Serra-Marques et al., 2020). Rajeevan et al. (2020) showed that the C-terminus NuMA^2058–2115^ fragment is sufficient to bind DNA *in vitro*, and the basic amino acids within the region are critical for its interaction with chromatin in cells (Figure 2C). When endogenous NuMA was replaced with mutated versions lacking its DNA-binding ability, cells showed improper chromosome decondensation during mitotic exit and an abnormal nuclear shape (Rajeevan et al., 2020), suggesting that NuMA–DNA interactions are critical for proper regulation of chromosome decondensation during nuclear reformation.

## Nuclear Localization Sequence/Signal in NuMA’s C-Terminal Region

NuMA has a nuclear localization signal/sequence (NLS) between the MTBDs in its C-terminal region (Tang et al., 1994; Figure 2C). When Chang et al. (2017) solved the crystal structure of the importin-α-NuMA-C-terminus complex, they found that NuMA–NLS exhibits a novel, non-classic interaction mode with importin-α, and that importin-β sterically inhibits NuMA’s MTBD2 *in vitro*. The NLS sequence is well conserved from *H. sapiens* to *X. laevis* (Chang et al., 2017), but not in *C. elegans* LIN-5, or *drosphila* Mud (Lorson et al., 2000; Siller et al., 2006). In fish, the highly conserved KR, H and KK residues of the NLS, which interact with the minor-, linker and the major-NLS-binding site on importin-α, respectively (Chang et al., 2017), are not identical and several amino acids are inserted between the H and KK residues (Tsuchiya et al., 2021); yet the Zebrafish NuMA C-terminal region can be targeted to the nucleus (Segalen et al., 2010). It would be interesting to understand whether NuMA localizes to the nucleus in other fish species, and why the NLS was acquired in vertebrates.

## The Clustering Domain in NuMA’s C-Terminal Region

The C-terminal globular domain has another key feature that facilitates NuMA’s self-assembly into oligomers (Harborth et al., 1999). *In vitro*, 10–12 NuMA homo-dimers assemble through its C-terminal region to form multi-arm oligomers. Each oligomer has a central clustered core with projected arms, and may be connected to create 3D nuclear architecture during interphase (Harborth et al., 1999). NuMA’s punctate signals at the cell cortex are most likely a result of its oligomerization/clustering. This clustering activity is attributed to a well-conserved 10 a.a sequence of NuMA^1768–1777^ (Okumura et al., 2018). Mutant analyses indicated that NuMA’s clustering is required for spindle pulling and spindle orientation at metaphase (Okumura et al., 2018), and for spindle bipolarization during prometaphase in acentrosomal human cells (Chinen et al., 2020), but is dispensable for spindle pole focusing (Okumura et al., 2018). It will be exciting to see what kind of structures are actually generated in cells by NuMA’s clustering activity both in mitosis and interphase. Especially, it is important to understand how this clustering activity synergetically functions with NuMA–LGN oligomer formation to organize high-ordered functional structures that capture and pull on astral microtubules at the mitotic cell cortex (Pirovano et al., 2019; Figure 3).

## Mitotic and Meiotic Regulation of NuMA

Since NuMA’s C-terminal domain binds to microtubules, the plasma membrane, and chromatin (Figure 2C), these interactions must be regulated throughout the cell cycle. In fact, the well conserved threonine at 2055 (T2055) is phosphorylated by CDK during metaphase (Compton and Luo, 1995; Kotak et al., 2013; Seldin et al., 2013), which inhibits NuMA’s cortical association and thus promotes microtubule-binding. CDK-based phosphorylation of NuMA is also critical for releasing NuMA from the chromosomes at mitotic entry (Rajeevan et al., 2020). During anaphase, NuMA is dephosphorylated by the PPP2CA–B55gamma–PPP2R1B complex (Keshri et al., 2020), which dissociates NuMA from the spindle poles (Gehmlich et al., 2004) and promotes its cortical association.

The microtubule binding activity of NuMA is vital for its mitotic and meiotic functions (Silk et al., 2009; Kolano et al., 2012; Tsuchiya et al., 2021). Thus, importins that sterically inhibit C-terminal microtubule-binding (Chang et al., 2017) must be released during mitosis. Previously, it was demonstrated that the NuMA-importin interaction is disrupted by Ran-GTP binding to importin-β in *Xenopus* egg extracts (Nachury et al., 2001; Wiese et al., 2001), and that Ran-GTP is essential for acentrosomal spindle assembly in female meiosis (Dumont et al., 2007; Holubcova et al., 2015; Drutovic et al., 2020). However, recent evidence suggests that Ran-GTP is not essential for mitotic spindle assembly and mitotic progression in chicken DT40 (Furuta et al., 2016) and human HCT116 cells (Tsuchiya et al., 2021). In addition, Ran-GTP is not required to activate NuMA and TPX2 in HCT116 cells (Tsuchiya et al., 2021), suggesting that additional parallel pathways exist to activate these proteins at a distance from chromosomes (Wei et al., 2015; Eibes et al., 2018; Brownlee and Heald, 2019). As cellular concentrations of Ran-GTP are variable across cell types and organisms (Kalab et al., 2006; Hasegawa et al., 2013), and the size of spindles and cells change dramatically during early embryonic divisions (Courtois et al., 2012; Levy and Heald, 2012), it would be important to determine how NuMA is spatiotemporally activated by Ran and other factors during mitosis and meiosis.

## Mitotic Regulation of NuMA at the Spindle Poles and the Cell Cortex

Once activated, the NuMA homo-dimer binds to microtubules independently of dynein (Heald et al., 1997; Hueschen et al., 2017) and cross-links two microtubules using its two C-terminal globular domains (Figure 3A; Forth et al., 2014). This microtubule crosslinking function may be facilitated at the spindle poles by binding to other microtubule associated proteins such as Rae1 (Wong et al., 2006), Eg5 (Iwakiri et al., 2013), and dynein/dynactin complexes (Merdes et al., 1996, 2000). On the other hand, when new microtubules are created in the spindle, NuMA is targeted to their minus-ends and subsequently forms a complex with dynein and dynactin; when this complex binds adjacent microtubules and moves toward their minus end, it pulls the NuMA-bound microtubules along, resulting in a focused spindle pole (Hueschen et al., 2017; Figure 3A).

At the poles, NuMA is phosphorylated by Aurora-A kinase at S1969, which leads to its dynamic mobility from the spindle poles to the cell cortex during metaphase (Gallini et al., 2016; Kotak et al., 2016; Figure 2C). NuMA is also phosphorylated by Polo-like kinase 1 (Plk1) at SS1833/34 (Kettenbach et al., 2011), which promotes NuMA’s turnover rate at both spindle poles and the cell cortex, and when inhibited results in NuMA’s accumulation at both locations (Sana et al., 2018). Plk1 localizes at the spindle poles, but also accumulates at kinetochores of misaligned chromosomes which locally diminish cortical LGN when they are located near the cell cortex (Tame et al., 2016). Since NuMA and LGN are inter-dependent (Du and Macara, 2004), the kinetochore-localized Plk1 may also target NuMA, which in turn reduces LGN. However, artificial membrane tethering of Plk1 dissociated dynein, but not LGN, from the cell cortex (Kiyomitsu and Cheeseman, 2012). In addition, when the immunoprecipitated GFP–LGN complexes were incubated with Plk1, Plk1 dissociated dynein and dynactin, but not NuMA, from LGN (Kiyomitsu and Cheeseman, 2012). Therefore, kinetochore- or centrosome-localized Plk1 may down-regulate the NuMA–LGN interaction synergetically in cooperation with other factors derived from chromosomes or centrosomes. Another kinase, ABL1, phosphorylates the well-conserved Y1774 residue of NuMA to control spindle orientation (Matsumura et al., 2012). Y1774 is located in the clustering motif (Okumura et al., 2018), but it remains unknown whether ABL1 regulates NuMA’s clustering activity.

In addition to these kinases, Ran-GTP gradients negatively regulate cortical NuMA–LGN localization near chromosomes in a distance dependent manner (Kiyomitsu and Cheeseman, 2012). Although molecular mechanisms by which Ran-GTP eliminates the NuMA–LGN complex from the cell cortex remain unclear, the cortical patterning created by chromosome-derived Ran-GTP is sufficient to explain why the mitotic spindle orients along its interphase cell axis (Dimitracopoulos et al., 2020). NuMA’s continuous exclusion from the equatorial region of the cell cortex during anaphase is dependent on the signals downstream of the centralspindlin complexes (Kotak et al., 2014).

## Interphase Function of NuMA

Several lines of evidence indicate that NuMA acts as a non-essential nucleoskeletal element in interphase (Zeng et al., 1994; Merdes and Cleveland, 1998; Harborth et al., 1999), which is nicely reviewed by Radulescu and Cleveland (2010). However, it is difficult to understand this role separately from the several roles it plays during mitosis since mitotic errors cause abnormal nuclei. Recently, Serra-Marques et al. (2020) nicely demonstrated that NuMA’s contribution to building a single, round nucleus is independent from its mitotic functions by inducing mitotic exit in NuMA KO Rpe1 cells without spindles (Figure 1B). Furthermore, they showed that NuMA keeps the decondensing chromosome mass compact during mitotic exit (Serra-Marques et al., 2020). Although the precise mechanisms are still unclear, evidence suggests that NuMA, like barrier-to-auto integration factor (BAF) at the chromosome ensemble surface (Samwer et al., 2017), may offer structural support throughout the nucleus by cross-linking chromosomes and preventing the nuclear envelop from penetrating into the chromosome mass, which results in a decrease of multinucleation and abnormally shaped nuclei during mitotic exit (Serra-Marques et al., 2020). Alternatively, NuMA may coordinate chromosome compaction and nuclear envelop assembly using its binding abilities to both chromosomes and membrane (Serra-Marques et al., 2020), as well as its binding to importins which can recruit membrane vesicles and nucleoporins (Lu et al., 2012).

In addition to its roles during mitotic exit, NuMA promotes the nucleus’ mechanical robustness (Serra-Marques et al., 2020) and could contribute to several interphase events, including chromatin organization (Abad et al., 2007), gene expression (Harborth et al., 2000; Ohata et al., 2013), DNA repair (Vidi et al., 2014; Moreno et al., 2019), and apoptosis (Gueth-Hallonet et al., 1997; Kivinen et al., 2005; Lin et al., 2007). Since chromatin architecture is highly dynamic during different phases of the cell cycle (Shoaib et al., 2020), acute protein depletion technologies, such as auxin-inducible degron (AID) (Natsume et al., 2016; Yesbolatova et al., 2020; Tsuchiya et al., 2021), would be useful to precisely understand the interphase functions of NuMA in future studies.

## Discussion

Vertebrate NuMA, and its related proteins in invertebrates, have been extensively studied using many techniques and model organisms. Over the past 10 years, much light has been shed on this multi-functional protein, revealing key domains, modifications and binding partners. However, we still do not know how NuMA contributes to spindle pole focusing, spindle positioning or nuclear formation at the molecular and structural level. In the next 10 years, it would be especially important to visualize the functional structures of NuMA and its complexes using biochemical reconstitution, high-resolution imaging and *in situ* structural analyses. In addition, most reported interactions and functions are not sufficiently validated in a physiological condition. Since many functions discussed in this review appear to be specific in vertebrates, vertebrate developmental models would be useful to obtain a comprehensive understanding of the diverse functions of NuMA. Furthermore, optogenetic manipulation of NuMA, or its related proteins, would serve as a powerful tool to control spindle position and orientation (Fielmich et al., 2018; Okumura et al., 2018), and could reveal physiological roles of division orientation and daughter cell size during development (Jankele et al., 2021).

Many key questions about NuMA remain to be answered. (1) How does NuMA recognize microtubule minus-ends and depolymerizing microtubule plus-ends? (2) How are dynein–dynactin–NuMA complexes formed during mitosis and meiosis and regulated at the spindle poles? (3) How are different cortical NuMA complexes assembled at the mitotic cell cortex and spatiotemporally regulated by intrinsic and extrinsic signals? (4) When does NuMA start to localize at the mitotic cell cortex to control spindle positioning during early vertebrate development? (5) What kinds of high-ordered structures are created by NuMA at the cell cortex and interphase nucleus to generate cortical spindle pulling forces and a mechanically robust nucleus, respectively? (6) How is NuMA dissociated from importins in a Ran-independent manner? (7) Why did NuMA acquire an NLS in vertebrates? (8) How has the NuMA gene evolved to achieve different functions in different organisms? Addressing these questions must provide new, exciting insights not only to advance our knowledge about nucleus formation, spindle assembly and spindle positioning, but also to understand how complex human cell architecture evolved.

## Author Contributions

TK wrote the first draft, acquired the fund. TK and SB revised manuscript, figures, and Table. SB got the images of Figure 1A. Both authors contributed to the article and approved the submitted version.

## Conflict of Interest

The authors declare that the research was conducted in the absence of any commercial or financial relationships that could be construed as a potential conflict of interest.

## References

[B1] Abad P. C. Lewis J. I Mian S. Knowles D. W. Sturgis J. Badve S. (2007). NuMA influences higher order chromatin organization in human mammary epithelium. *Mol. Biol. Cell* 18 348–361. 10.1091/mbc.e06-06-0551 17108325 PMC1783787

[B2] Alvarez Sedo C. Schatten H. Combelles C. M. Rawe V. Y. (2011). The nuclear mitotic apparatus (NuMA) protein: localization and dynamics in human oocytes, fertilization and early embryos. *Mol. Hum. Reprod*. 17 392–398. 10.1093/molehr/gar009 21297155

[B3] Baines A. J. Lu H. C. Bennett P. M. (2014). The Protein 4.1 family: hub proteins in animals for organizing membrane proteins. *Biochim. Biophys. Acta* 1838 605–619. 10.1016/j.bbamem.2013.05.030 23747363

[B4] Borgal L. Wakefield J. G. (2018). Context-dependent spindle pole focusing. *Essays Biochem*. 62 803–813. 10.1042/ebc20180034 30429281

[B5] Bowman S. K. Neumuller R. A. Novatchkova M. Du Q. Knoblich J. A. (2006). The *Drosophila* NuMA homolog mud regulates spindle orientation in asymmetric cell division. *Dev. Cell* 10 731–742. 10.1016/j.devcel.2006.05.005 16740476

[B6] Brownlee C. Heald R. (2019). Importin alpha partitioning to the plasma membrane regulates intracellular scaling. *Cell* 176 805–815.e808. 30639102 10.1016/j.cell.2018.12.001 PMC6368448

[B7] Carminati M. Gallini S. Pirovano L. Alfieri A. Bisi S. Mapelli M. (2016). Concomitant binding of Afadin to LGN and F-actin directs planar spindle orientation. *Nat. Struct. Mol. Biol*. 23 155–163. 10.1038/nsmb.3152 26751642

[B8] Chang C. C. Huang T. L. Shimamoto Y. Tsai S. Y. Hsia K. C. (2017). Regulation of mitotic spindle assembly factor NuMA by Importin-beta. *J. Cell Biol*. 216 3453–3462. 10.1083/jcb.201705168 28939615 PMC5674899

[B9] Chinen T. Yamamoto S. Takeda Y. Watanabe K. Kuroki K. Hashimoto K. (2020). NuMA assemblies organize microtubule asters to establish spindle bipolarity in acentrosomal human cells. *EMBO J*. 39:e102378. 10.15252/embj.2019102378 PMC6960446 31782546

[B10] Cleveland D. W. (1995). NuMA: a protein involved in nuclear structure, spindle assembly, and nuclear re-formation. *Trends Cell Biol*. 5 60–64. 10.1016/s0962-8924(00)88947-3 14731413

[B11] Collins E. S. Balchand S. K. Faraci J. L. Wadsworth P. Lee W. L. (2012). Cell cycle-regulated cortical dynein/dynactin promotes symmetric cell division by differential pole motion in anaphase. *Mol. Biol. Cell* 23 3380–3390. 10.1091/mbc.e12-02-0109 22809624 PMC3431930

[B12] Compton D. A. Cleveland D. W. (1994). NuMA, a nuclear protein involved in mitosis and nuclear reformation. *Curr. Opin. Cell Biol*. 6 343–346. 10.1016/0955-0674(94)90024-8 7917323

[B13] Compton D. A. Luo C. (1995). Mutation of the predicted p34cdc2 phosphorylation sites in NuMA impair the assembly of the mitotic spindle and block mitosis. *J. Cell Sci*. 108(Pt 2) 621–633. 7769006 10.1242/jcs.108.2.621

[B14] Compton D. A. Szilak I. Cleveland D. W. (1992). Primary structure of NuMA, an intranuclear protein that defines a novel pathway for segregation of proteins at mitosis. *J. Cell Biol*. 116 1395–1408. 10.1083/jcb.116.6.1395 1541636 PMC2289377

[B15] Courtois A. Schuh M. Ellenberg J. Hiiragi T. (2012). The transition from meiotic to mitotic spindle assembly is gradual during early mammalian development. *J. Cell Biol*. 198 357–370. 10.1083/jcb.201202135 22851319 PMC3413348

[B16] Culurgioni S. Alfieri A. Pendolino V. Laddomada F. Mapelli M. (2011). Inscuteable and NuMA proteins bind competitively to Leu-Gly-Asn repeat-enriched protein (LGN) during asymmetric cell divisions. *Proc. Natl. Acad. Sci. U.S.A*. 108 20998–21003. 10.1073/pnas.1113077108 22171003 PMC3248549

[B17] Culurgioni S. Mari S. Bonetti P. Gallini S. Bonetto G. Brennich M. (2018). Insc:LGN tetramers promote asymmetric divisions of mammary stem cells. *Nat. Commun*. 9:1025. 10.1038/s41467-018-03343-4 PMC5844954 29523789

[B18] Dimitracopoulos A. Srivastava P. Chaigne A. Win Z. Shlomovitz R. Lancaster O. M. (2020). Mechanochemical crosstalk produces cell-intrinsic patterning of the cortex to orient the mitotic spindle. *Curr. Biol*. 30 3687–3696.e3684. 32735816 10.1016/j.cub.2020.06.098 PMC7521479

[B19] Drutovic D. Duan X. Li R. Kalab P. Solc P. (2020). RanGTP and importin beta regulate meiosis I spindle assembly and function in mouse oocytes. *EMBO J*. 39:e101689. 10.15252/embj.2019101689 PMC6939199 31617608

[B20] Du Q. Macara I. G. (2004). Mammalian Pins is a conformational switch that links NuMA to heterotrimeric G proteins. *Cell* 119 503–516. 10.1016/j.cell.2004.10.028 15537540

[B21] Du Q. Taylor L. Compton D. A. Macara I. G. (2002). LGN blocks the ability of NuMA to bind and stabilize microtubules. A mechanism for mitotic spindle assembly regulation. *Curr. Biol*. 12 1928–1933. 10.1016/s0960-9822(02)01298-8 12445386

[B22] Dumont J. Petri S. Pellegrin F. Terret M. E. Bohnsack M. T. Rassinier P. (2007). A centriole- and RanGTP-independent spindle assembly pathway in meiosis I of vertebrate oocytes. *J. Cell Biol*. 176 295–305. 10.1083/jcb.200605199 17261848 PMC2063956

[B23] Eibes S. Gallisa-Sune N. Rosas-Salvans M. Martinez-Delgado P. Vernos I. Roig J. (2018). Nek9 phosphorylation defines a new role for TPX2 in Eg5-dependent centrosome separation before nuclear envelope breakdown. *Curr. Biol*. 28 121–129.e124. 29276125 10.1016/j.cub.2017.11.046

[B24] Fielmich L. E. Schmidt R. Dickinson D. J. Goldstein B. Akhmanova A. van den Heuvel S. (2018). Optogenetic dissection of mitotic spindle positioning in vivo. *Elife* 7:e38198. 10.7554/eLife.38198 PMC6214656 30109984

[B25] Forth S. Hsia K. C. Shimamoto Y. Kapoor T. M. (2014). Asymmetric friction of nonmotor MAPs can lead to their directional motion in active microtubule networks. *Cell* 157 420–432. 10.1016/j.cell.2014.02.018 24725408 PMC4015189

[B26] Furuta M. Hori T. Fukagawa T. (2016). Chromatin binding of RCC1 during mitosis is important for its nuclear localization in interphase. *Mol. Biol. Cell* 27 371–381. 10.1091/mbc.e15-07-0497 26564799 PMC4713138

[B27] Galli M. van den Heuvel S. (2008). Determination of the cleavage plane in early C. elegans embryos. *Annu. Rev. Genet*. 42 389–411. 10.1146/annurev.genet.40.110405.090523 18710303

[B28] Gallini S. Carminati M. De Mattia F. Pirovano L. Martini E. Oldani A. I (2016). NuMA phosphorylation by aurora-A orchestrates spindle orientation. *Curr. Biol*. 26 458–469. 10.1016/j.cub.2015.12.051 26832443

[B29] Gama J. B. Pereira C. Simoes P. A. Celestino R. Reis R. M. Barbosa D. J. (2017). Molecular mechanism of dynein recruitment to kinetochores by the Rod-Zw10-Zwilch complex and Spindly. *J. Cell Biol*. 216 943–960. 10.1083/jcb.201610108 28320824 PMC5379953

[B30] Gehmlich K. Haren L. Merdes A. (2004). Cyclin B degradation leads to NuMA release from dynein/dynactin and from spindle poles. *EMBO Rep*. 5 97–103. 10.1038/sj.embor.7400046 14710193 PMC1298957

[B31] Gonczy P. (2008). Mechanisms of asymmetric cell division: flies and worms pave the way. *Nat. Rev. Mol. Cell Biol*. 9 355–366. 10.1038/nrm2388 18431399

[B32] Greenberg S. R. Tan W. Lee W. L. (2018). Num1 versus NuMA: insights from two functionally homologous proteins. *Biophys. Rev*. 10 1631–1636. 10.1007/s12551-018-0472-x 30402673 PMC6297085

[B33] Gueth-Hallonet C. Wang J. Harborth J. Weber K. Osborn M. (1998). Induction of a regular nuclear lattice by overexpression of NuMA. *Exp. Cell Res*. 243 434–452. 10.1006/excr.1998.4178 9743603

[B34] Gueth-Hallonet C. Weber K. Osborn M. (1997). Cleavage of the nuclear matrix protein NuMA during apoptosis. *Exp. Cell Res*. 233 21–24. 10.1006/excr.1997.3557 9184071

[B35] Harborth J. Wang J. Gueth-Hallonet C. Weber K. Osborn M. (1999). Self assembly of NuMA: multiarm oligomers as structural units of a nuclear lattice. *EMBO J*. 18 1689–1700. 10.1093/emboj/18.6.1689 10075938 PMC1171255

[B36] Harborth J. Weber K. Osborn M. (1995). Epitope mapping and direct visualization of the parallel, in-register arrangement of the double-stranded coiled-coil in the NuMA protein. *EMBO J*. 14 2447–2460. 10.1002/j.1460-2075.1995.tb07242.x 7781599 PMC398358

[B37] Harborth J. Weber K. Osborn M. (2000). GAS41, a highly conserved protein in eukaryotic nuclei, binds to NuMA. *J. Biol. Chem*. 275 31979–31985. 10.1074/jbc.m000994200 10913114

[B38] Hasegawa K. Ryu S. J. Kalab P. (2013). Chromosomal gain promotes formation of a steep RanGTP gradient that drives mitosis in aneuploid cells. *J. Cell Biol*. 200 151–161. 10.1083/jcb.201206142 23319601 PMC3549973

[B39] Heald R. Tournebize R. Habermann A. Karsenti E. Hyman A. (1997). Spindle assembly in Xenopus egg extracts: respective roles of centrosomes and microtubule self-organization. *J. Cell Biol*. 138 615–628. 10.1083/jcb.138.3.615 9245790 PMC2141625

[B40] Holubcova Z. Blayney M. Elder K. Schuh M. (2015). Human oocytes. Error-prone chromosome-mediated spindle assembly favors chromosome segregation defects in human oocytes. *Science* 348 1143–1147. 10.1126/science.aaa9529 26045437 PMC4477045

[B41] Hueschen C. L. Kenny S. J. Xu K. Dumont S. (2017). NuMA recruits dynein activity to microtubule minus-ends at mitosis. *Elife* 6:e29328. 10.7554/eLife.29328 PMC5706958 29185983

[B42] Iwakiri Y. Kamakura S. Hayase J. Sumimoto H. (2013). Interaction of NuMA protein with the kinesin Eg5: its possible role in bipolar spindle assembly and chromosome alignment. *Biochem. J*. 451 195–204. 10.1042/bj20121447 23368718

[B43] Izumi Y. Ohta N. Hisata K. Raabe T. Matsuzaki F. (2006). *Drosophila* Pins-binding protein Mud regulates spindle-polarity coupling and centrosome organization. *Nat. Cell Biol*. 8 586–593. 10.1038/ncb1409 16648846

[B44] Jankele R. Jelier R. Gonczy P. (2021). Physically asymmetric division of the C. elegans zygote ensures invariably successful embryogenesis. *Elife* 10:e61714. 10.7554/eLife.61714 PMC7972452 33620314

[B45] Jia M. Li J. Zhu J. Wen W. Zhang M. Wang W. (2012). Crystal structures of the scaffolding protein LGN reveal the general mechanism by which GoLoco binding motifs inhibit the release of GDP from Galphai. *J. Biol. Chem*. 287 36766–36776. 10.1074/jbc.m112.391607 22952234 PMC3481280

[B46] Kaji N. Muramoto A. Mizuno K. (2008). LIM kinase-mediated cofilin phosphorylation during mitosis is required for precise spindle positioning. *J. Biol. Chem*. 283 4983–4992. 10.1074/jbc.m708644200 18079118

[B47] Kalab P. Pralle A. Isacoff E. Y. Heald R. Weis K. (2006). Analysis of a RanGTP-regulated gradient in mitotic somatic cells. *Nature* 440 697–701. 10.1038/nature04589 16572176

[B48] Keshri R. Rajeevan A. Kotak S. (2020). PP2A–B55gamma counteracts Cdk1 and regulates proper spindle orientation through the cortical dynein adaptor NuMA. *J. Cell Sci*. 133:jcs243857. 10.1242/jcs.243857 32591484 PMC7406356

[B49] Kettenbach A. N. Schweppe D. K. Faherty B. K. Pechenick D. Pletnev A. A. Gerber S. A. (2011). Quantitative phosphoproteomics identifies substrates and functional modules of Aurora and Polo-like kinase activities in mitotic cells. *Sci. Signal*. 4:rs5. 10.1126/scisignal.2001497 21712546 PMC3808085

[B50] Kivinen K. Kallajoki M. Taimen P. (2005). Caspase-3 is required in the apoptotic disintegration of the nuclear matrix. *Exp. Cell Res*. 311 62–73. 10.1016/j.yexcr.2005.08.006 16199031

[B51] Kiyomitsu T. (2019). The cortical force-generating machinery: how cortical spindle-pulling forces are generated. *Curr. Opin. Cell Biol*. 60 1–8. 10.1016/j.ceb.2019.03.001 30954860

[B52] Kiyomitsu T. Cheeseman I. M. (2012). Chromosome- and spindle-pole-derived signals generate an intrinsic code for spindle position and orientation. *Nat. Cell Biol*. 14 311–317. 10.1038/ncb2440 22327364 PMC3290711

[B53] Kiyomitsu T. Cheeseman I. M. (2013). Cortical dynein and asymmetric membrane elongation coordinately position the spindle in anaphase. *Cell* 154 391–402. 10.1016/j.cell.2013.06.010 23870127 PMC4177044

[B54] Kolano A. Brunet S. Silk A. D. Cleveland D. W. Verlhac M. H. (2012). Error-prone mammalian female meiosis from silencing the spindle assembly checkpoint without normal interkinetochore tension. *Proc. Natl. Acad. Sci. U.S.A*. 109 E1858–E1867. 22552228 10.1073/pnas.1204686109 PMC3390881

[B55] Kotak S. Afshar K. Busso C. Gonczy P. (2016). Aurora A kinase regulates proper spindle positioning in C. elegans and in human cells. *J. Cell Sci*. 129 3015–3025. 10.1242/jcs.184416 27335426 PMC6203311

[B56] Kotak S. Busso C. Gonczy P. (2012). Cortical dynein is critical for proper spindle positioning in human cells. *J. Cell Biol*. 199 97–110. 10.1083/jcb.201203166 23027904 PMC3461507

[B57] Kotak S. Busso C. Gonczy P. (2013). NuMA phosphorylation by CDK1 couples mitotic progression with cortical dynein function. *EMBO J*. 32 2517–2529. 10.1038/emboj.2013.172 23921553 PMC3770949

[B58] Kotak S. Busso C. Gonczy P. (2014). NuMA interacts with phosphoinositides and links the mitotic spindle with the plasma membrane. *EMBO J*. 33 1815–1830. 10.15252/embj.201488147 24996901 PMC4195763

[B59] Kschonsak Y. T. Hoffmann I. (2018). Activated ezrin controls MISP levels to ensure correct NuMA polarization and spindle orientation. *J. Cell Sci*. 131:jcs214544. 10.1242/jcs.214544 29669740

[B60] Lechler T. Fuchs E. (2005). Asymmetric cell divisions promote stratification and differentiation of mammalian skin. *Nature* 437 275–280. 10.1038/nature03922 16094321 PMC1399371

[B61] Lee I. G. Cason S. E. Alqassim S. S. Holzbaur E. L. F. Dominguez R. (2020). A tunable LIC1-adaptor interaction modulates dynein activity in a cargo-specific manner. *Nat. Commun*. 11:5695. 10.1038/s41467-020-19538-7 PMC7655957 33173051

[B62] Levy D. L. Heald R. (2012). Mechanisms of intracellular scaling. *Annu. Rev. Cell Dev. Biol*. 28 113–135. 10.1146/annurev-cellbio-092910-154158 22804576

[B63] Lin H. H. Hsu H. L. Yeh N. H. (2007). Apoptotic cleavage of NuMA at the C-terminal end is related to nuclear disruption and death amplification. *J. Biomed. Sci*. 14 681–694. 10.1007/s11373-007-9165-3 17401638

[B64] Lorson M. A. Horvitz H. R. van den Heuvel S. (2000). LIN-5 is a novel component of the spindle apparatus required for chromosome segregation and cleavage plane specification in Caenorhabditis elegans. *J. Cell Biol*. 148 73–86. 10.1083/jcb.148.1.73 10629219 PMC3207147

[B65] Lu Q. Lu Z. Liu Q. Guo L. Ren H. Fu J. (2012). Chromatin-bound NLS proteins recruit membrane vesicles and nucleoporins for nuclear envelope assembly via importin-alpha/beta. *Cell Res*. 22 1562–1575. 10.1038/cr.2012.113 22847741 PMC3494395

[B66] Luderus M. E. den Blaauwen J. L. de Smit O. J. Compton D. A. van Driel R. (1994). Binding of matrix attachment regions to lamin polymers involves single-stranded regions and the minor groove. *Mol. Cell. Biol*. 14 6297–6305. 10.1128/mcb.14.9.6297 8065361 PMC359156

[B67] Lydersen B. K. Kao F. T. Pettijohn D. (1980). Expression of genes coding for non-histone chromosomal proteins in human-Chinese hamster cell hybrids. An electrophoretic analysis. *J. Biol. Chem*. 255 3002–3007. 10.1016/s0021-9258(19)85842-8 7358723

[B68] Lydersen B. K. Pettijohn D. E. (1980). Human-specific nuclear protein that associates with the polar region of the mitotic apparatus: distribution in a human/hamster hybrid cell. *Cell* 22 489–499. 10.1016/0092-8674(80)90359-1 7004645

[B69] Machicoane M. de Frutos C. A. Fink J. Rocancourt M. Lombardi Y. Garel S. (2014). SLK-dependent activation of ERMs controls LGN-NuMA localization and spindle orientation. *J. Cell Biol*. 205 791–799. 10.1083/jcb.201401049 24958772 PMC4068135

[B70] Matsumura S. Hamasaki M. Yamamoto T. Ebisuya M. Sato M. Nishida E. (2012). ABL1 regulates spindle orientation in adherent cells and mammalian skin. *Nat. Commun*. 3:626. 10.1038/ncomms1634 PMC3324324 22252550

[B71] Mattagajasingh S. N. Huang S. C. Benz E. J. Jr. (2009). Inhibition of protein 4.1 R and NuMA interaction by mutagenization of their binding-sites abrogates nuclear localization of 4.1 R. *Clin. Transl. Sci*. 2 102–111. 10.1111/j.1752-8062.2008.00087.x 20443879 PMC5350677

[B72] McKenney R. J. Huynh W. Tanenbaum M. E. Bhabha G. Vale R. D. (2014). Activation of cytoplasmic dynein motility by dynactin-cargo adapter complexes. *Science* 345 337–341. 10.1126/science.1254198 25035494 PMC4224444

[B73] Merdes A. Cleveland D. W. (1998). The role of NuMA in the interphase nucleus. *J. Cell Sci*. 111(Pt 1) 71–79. 9394013 10.1242/jcs.111.1.71

[B74] Merdes A. Heald R. Samejima K. Earnshaw W. C. Cleveland D. W. (2000). Formation of spindle poles by dynein/dynactin-dependent transport of NuMA. *J. Cell Biol*. 149 851–862. 10.1083/jcb.149.4.851 10811826 PMC2174573

[B75] Merdes A. Ramyar K. Vechio J. D. Cleveland D. W. (1996). A complex of NuMA and cytoplasmic dynein is essential for mitotic spindle assembly. *Cell* 87 447–458. 10.1016/s0092-8674(00)81365-3 8898198

[B76] Moreno N. S. Liu J. Haas K. M. Parker L. L. Chakraborty C. Kron S. J. (2019). The nuclear structural protein NuMA is a negative regulator of 53BP1 in DNA double-strand break repair. *Nucleic Acids Res*. 47 10475. 10.1093/nar/gkz802 31511892 PMC6821149

[B77] Morin X. Bellaiche Y. (2011). Mitotic spindle orientation in asymmetric and symmetric cell divisions during animal development. *Dev. Cell* 21 102–119. 10.1016/j.devcel.2011.06.012 21763612

[B78] Nachury M. V. Maresca T. J. Salmon W. C. Waterman-Storer C. M. Heald R. Weis K. (2001). Importin beta is a mitotic target of the small GTPase Ran in spindle assembly. *Cell* 104 95–106. 10.1016/s0092-8674(01)00194-5 11163243

[B79] Natsume T. Kiyomitsu T. Saga Y. Kanemaki M. T. (2016). Rapid protein depletion in human cells by auxin-inducible degron tagging with short homology donors. *Cell Rep*. 15 210–218. 10.1016/j.celrep.2016.03.001 27052166

[B80] Ohata H. Miyazaki M. Otomo R. Matsushima-Hibiya Y. Otsubo C. Nagase T. (2013). NuMA is required for the selective induction of p53 target genes. *Mol. Cell. Biol*. 33 2447–2457. 10.1128/mcb.01221-12 23589328 PMC3700099

[B81] Okumura M. Natsume T. Kanemaki M. T. Kiyomitsu T. (2018). Dynein-Dynactin-NuMA clusters generate cortical spindle-pulling forces as a multi-arm ensemble. *Elife* 7:e36559. 10.7554/eLife.36559 PMC6037482 29848445

[B82] Peyre E. Jaouen F. Saadaoui M. Haren L. Merdes A. Durbec P. (2011). A lateral belt of cortical LGN and NuMA guides mitotic spindle movements and planar division in neuroepithelial cells. *J. Cell Biol*. 193 141–154. 10.1083/jcb.201101039 21444683 PMC3082188

[B83] Pirovano L. Culurgioni S. Carminati M. Alfieri A. Monzani S. Cecatiello V. (2019). Hexameric NuMA:LGN structures promote multivalent interactions required for planar epithelial divisions. *Nat. Commun*. 10:2208. 10.1038/s41467-019-09999-w PMC6525239 31101817

[B84] Poulson N. D. Lechler T. (2010). Robust control of mitotic spindle orientation in the developing epidermis. *J. Cell Biol*. 191 915–922. 10.1083/jcb.201008001 21098114 PMC2995176

[B85] Radulescu A. E. Cleveland D. W. (2010). NuMA after 30 years: the matrix revisited. *Trends Cell Biol*. 20 214–222. 10.1016/j.tcb.2010.01.003 20137953 PMC3137513

[B86] Rajeevan A. Keshri R. Kapoor S. Kotak S. (2020). NuMA interaction with chromatin is vital for proper chromosome decondensation at the mitotic exit. *Mol. Biol. Cell* 31 2437–2451. 10.1091/mbc.e20-06-0415 32845810 PMC7851854

[B87] Renna C. Rizzelli F. Carminati M. Gaddoni C. Pirovano L. Cecatiello V. (2020). Organizational principles of the NuMA-dynein interaction interface and implications for mitotic spindle functions. *Structure* 28 820–829.e826. 32413290 10.1016/j.str.2020.04.017

[B88] Saadaoui M. Machicoane M. di Pietro F. Etoc F. Echard A. Morin X. (2014). Dlg1 controls planar spindle orientation in the neuroepithelium through direct interaction with LGN. *J. Cell Biol*. 206 707–717. 10.1083/jcb.201405060 25202028 PMC4164945

[B89] Samwer M. Schneider M. W. G. Hoefler R. Schmalhorst P. S. Jude J. G. Zuber J. (2017). DNA cross-bridging shapes a single nucleus from a set of mitotic chromosomes. *Cell* 170 956–972.e923. 28841419 10.1016/j.cell.2017.07.038 PMC5638020

[B90] Sana S. Keshri R. Rajeevan A. Kapoor S. Kotak S. (2018). Plk1 regulates spindle orientation by phosphorylating NuMA in human cells. *Life Sci. Alliance* 1:e201800223. 10.26508/lsa.201800223 30456393 PMC6240335

[B91] Schlager M. A. Hoang H. T. Urnavicius L. Bullock S. L. Carter A. P. (2014). In vitro reconstitution of a highly processive recombinant human dynein complex. *EMBO J*. 33 1855–1868. 10.15252/embj.201488792 24986880 PMC4158905

[B92] Segalen M. Johnston C. A. Martin C. A. Dumortier J. G. Prehoda K. E. David N. B. (2010). The Fz-Dsh planar cell polarity pathway induces oriented cell division via Mud/NuMA in *Drosophila* and zebrafish. *Dev. Cell* 19 740–752. 10.1016/j.devcel.2010.10.004 21074723 PMC3008569

[B93] Seldin L. Muroyama A. Lechler T. (2016). NuMA-microtubule interactions are critical for spindle orientation and the morphogenesis of diverse epidermal structures. *Elife* 5:e12504. 10.7554/eLife.12504 PMC4758947 26765568

[B94] Seldin L. Poulson N. D. Foote H. P. Lechler T. (2013). NuMA localization, stability, and function in spindle orientation involve 4.1 and Cdk1 interactions. *Mol. Biol. Cell* 24 3651–3662. 10.1091/mbc.e13-05-0277 24109598 PMC3842992

[B95] Serra-Marques A. Houtekamer R. Hintzen D. Canty J. T. Yildiz A. Dumont S. (2020). The mitotic protein NuMA plays a spindle-independent role in nuclear formation and mechanics. *J. Cell Biol*. 219:e202004202. 10.1083/jcb.202004202 PMC7555356 33044554

[B96] Shoaib M. Nair N. Sorensen C. S. (2020). Chromatin landscaping at mitotic exit orchestrates genome function. *Front. Genet*. 11:103. 10.3389/fgene.2020.00103 32158468 PMC7052122

[B97] Silk A. D. Holland A. J. Cleveland D. W. (2009). Requirements for NuMA in maintenance and establishment of mammalian spindle poles. *J. Cell Biol*. 184 677–690. 10.1083/jcb.200810091 19255246 PMC2686415

[B98] Siller K. H. Cabernard C. Doe C. Q. (2006). The NuMA-related Mud protein binds Pins and regulates spindle orientation in *Drosophila* neuroblasts. *Nat. Cell Biol*. 8 594–600. 10.1038/ncb1412 16648843

[B99] Siller K. H. Doe C. Q. (2009). Spindle orientation during asymmetric cell division. *Nat. Cell Biol*. 11 365–374. 10.1038/ncb0409-365 19337318

[B100] Srinivasan D. G. Fisk R. M. Xu H. van den Heuvel S. (2003). A complex of LIN-5 and GPR proteins regulates G protein signaling and spindle function in C elegans. *Genes Dev*. 17 1225–1239. 10.1101/gad.1081203 12730122 PMC196055

[B101] Sun Q. Y. Schatten H. (2006). Role of NuMA in vertebrate cells: review of an intriguing multifunctional protein. *Front. Biosci*. 11:1137–1146. 10.2741/1868 16146802

[B102] Suzuki M. (1989). SPXX, a frequent sequence motif in gene regulatory proteins. *J. Mol. Biol*. 207 61–84. 10.1016/0022-2836(89)90441-5 2500531

[B103] Taimen P. Parvinen M. Osborn M. Kallajoki M. (2004). NuMA in rat testis–evidence for roles in proliferative activity and meiotic cell division. *Exp. Cell Res*. 298 512–520. 10.1016/j.yexcr.2004.05.002 15265698

[B104] Tame M. A. Raaijmakers J. A. Afanasyev P. Medema R. H. (2016). Chromosome misalignments induce spindle-positioning defects. *EMBO Rep*. 17 317–325. 10.15252/embr.201541143 26882550 PMC4772978

[B105] Tang T. K. Tang C. J. Chao Y. J. Wu C. W. (1994). Nuclear mitotic apparatus protein (NuMA): spindle association, nuclear targeting and differential subcellular localization of various NuMA isoforms. *J. Cell Sci*. 107(Pt 6) 1389–1402. 7962183 10.1242/jcs.107.6.1389

[B106] Tsuchiya K. Hayashi H. Nishina M. Okumura M. Sato Y. Kanemaki M. T. (2021). Ran-GTP is non-essential to activate NuMA for mitotic spindle-pole focusing but dynamically polarizes HURP near chromosomes. *Curr. Biol*. 31 115–127.e113. 33186548 10.1016/j.cub.2020.09.091

[B107] Vidi P. A. Liu J. Salles D. Jayaraman S. Dorfman G. Gray M. (2014). NuMA promotes homologous recombination repair by regulating the accumulation of the ISWI ATPase SNF2h at DNA breaks. *Nucleic Acids Res*. 42 6365–6379. 10.1093/nar/gku296 24753406 PMC4041463

[B108] Wei J. H. Zhang Z. C. Wynn R. M. Seemann J. (2015). GM130 regulates golgi-derived spindle assembly by activating TPX2 and capturing microtubules. *Cell* 162 287–299. 10.1016/j.cell.2015.06.014 26165940 PMC4506739

[B109] Wiese C. Wilde A. Moore M. S. Adam S. A. Merdes A. Zheng Y. (2001). Role of importin-beta in coupling Ran to downstream targets in microtubule assembly. *Science* 291 653–656. 10.1126/science.1057661 11229403

[B110] Williams S. E. Beronja S. Pasolli H. A. Fuchs E. (2011). Asymmetric cell divisions promote Notch-dependent epidermal differentiation. *Nature* 470 353–358. 10.1038/nature09793 21331036 PMC3077085

[B111] Wong R. W. Blobel G. Coutavas E. (2006). Rae1 interaction with NuMA is required for bipolar spindle formation. *Proc. Natl. Acad. Sci. U.S.A*. 103 19783–19787. 10.1073/pnas.0609582104 17172455 PMC1750899

[B112] Woodard G. E. Huang N. N. Cho H. Miki T. Tall G. G. Kehrl J. H. (2010). Ric-8A and Gi alpha recruit LGN, NuMA, and dynein to the cell cortex to help orient the mitotic spindle. *Mol. Cell. Biol*. 30 3519–3530. 10.1128/mcb.00394-10 20479129 PMC2897540

[B113] Yamada M. Goshima G. (2017). Mitotic spindle assembly in land plants: molecules and mechanisms. *Biology (Basel)* 6:6. 10.3390/biology6010006 28125061 PMC5371999

[B114] Yang C. H. Lambie E. J. Snyder M. (1992). NuMA: an unusually long coiled-coil related protein in the mammalian nucleus. *J. Cell Biol*. 116 1303–1317. 10.1083/jcb.116.6.1303 1541630 PMC2289379

[B115] Yesbolatova A. Saito Y. Kitamoto N. Makino-Itou H. Ajima R. Nakano R. (2020). The auxin-inducible degron 2 technology provides sharp degradation control in yeast, mammalian cells, and mice. *Nat. Commun*. 11:5701. 10.1038/s41467-020-19532-z PMC7659001 33177522

[B116] Yuzawa S. Kamakura S. Iwakiri Y. Hayase J. Sumimoto H. (2011). Structural basis for interaction between the conserved cell polarity proteins Inscuteable and Leu-Gly-Asn repeat-enriched protein (LGN). *Proc. Natl. Acad. Sci. U.S.A*. 108 19210–19215. 10.1073/pnas.1110951108 22074847 PMC3228485

[B117] Zeng C. He D. Brinkley B. R. (1994). Localization of NuMA protein isoforms in the nuclear matrix of mammalian cells. *Cell Motil. Cytoskeleton* 29 167–176. 10.1002/cm.970290208 7820866

[B118] Zheng Z. Wan Q. Meixiong G. Du Q. (2014). Cell cycle-regulated membrane binding of NuMA contributes to efficient anaphase chromosome separation. *Mol. Biol. Cell* 25 606–619. 10.1091/mbc.e13-08-0474 24371089 PMC3937087

[B119] Zhu J. Shang Y. Xia C. Wang W. Wen W. Zhang M. (2011a). Guanylate kinase domains of the MAGUK family scaffold proteins as specific phospho-protein-binding modules. *EMBO J*. 30 4986–4997. 10.1038/emboj.2011.428 22117215 PMC3243629

[B120] Zhu J. Wen W. Zheng Z. Shang Y. Wei Z. Xiao Z. (2011b). LGN/mInsc and LGN/NuMA complex structures suggest distinct functions in asymmetric cell division for the Par3/mInsc/LGN and Galphai/LGN/NuMA pathways. *Mol. Cell* 43 418–431. 10.1016/j.molcel.2011.07.011 21816348 PMC3158460

